# Severe paediatric conditions linked with EV-A71 and EV-D68, France, May to October 2016

**DOI:** 10.2807/1560-7917.ES.2016.21.46.30402

**Published:** 2016-11-17

**Authors:** Denise Antona, Manoëlle Kossorotoff, Isabelle Schuffenecker, Audrey Mirand, Marianne Leruez-Ville, Clément Bassi, Mélodie Aubart, Florence Moulin, Daniel Lévy-Bruhl, Cécile Henquell, Bruno Lina, Isabelle Desguerre

**Affiliations:** 1 Direction des maladies infectieuses, Santé publique France, Saint-Maurice, France; 2 Service de neuropédiatrie, AP-HP, Hôpital Necker – Enfants malades, Paris, France; 3 CNR des entérovirus et parechovirus, laboratoire de virologie, Hospices civils de Lyon, Lyon, France; 4 CNR des entérovirus et parechovirus-laboratoire associé, laboratoire de virologie, CHU de Clermont-Ferrand, Clermont Ferrand, France; 5 Laboratoire de virologie, AP-HP, Hôpital Necker – Enfants malades, Paris, France; 6 Cellule d’intervention en région Ile de France, Santé publique France, Paris, France; 7 Service de réanimation pédiatrique, AP-HP, Hôpital Necker – Enfants malades, Paris, France

**Keywords:** Enterovirus A71, Enterovirus D68, severe neurological symptoms, children, France

## Abstract

We report 59 cases of severe paediatric conditions linked with enterovirus (EV)-A71 and EV-D68 in France between May and October 2016. Fifty-two children had severe neurological symptoms. EV sequence-based typing for 42 cases revealed EV-A71 in 21 (18 subgenotype C1, detected for the first time in France) and EV-D68 in eight. Clinicians should be encouraged to obtain stool and respiratory specimens from patients presenting with severe neurological disorders for EV detection and characterisation.

On 29 July 2016, via an Early Warning and Response System message, French health authorities informed public health authorities in European countries about a recent increase of severe acute neurological conditions reported by one of the main academic paediatric hospitals in Paris [1]. A first local retrospective survey showed that, since April 2016, 18 children presented with rhombencephalitis, encephalitis, cerebellitis or myelitis and additional four with facial nerve radiculitis. Enterovirus (EV) infection was confirmed in eight of the 22 cases. Case finding was rapidly implemented at a national level. All paediatric wards (including neurology, paediatric intensive care units, internal medicine and emergency wards) were invited to report any case with severe conditions e.g. neurological, cardiac, neonatal sepsis, with suspected association with EV infections, starting from 15 March 2016, onwards. For suspected cases, they were requested to collect clinical specimens including cerebrospinal fluid (CSF), nasopharyngeal aspirates, stools and/or blood specimens for EV testing, and typing if positive for EV genome detection. Simultaneously, the two EV National Reference Laboratories (NRLs) in Clermont-Ferrand and Lyon, notified all laboratories participating in the French EV surveillance network (RSE) [2], who were already aware of the EV-A71 outbreak in Catalonia, Spain, since June 2016 [3]. Although the focus was on paediatric cases, a message was also sent to neurologists, internists and intensivists treating adult patients and participating in a prospective cohort study on encephalitis in adults (ENCEIF survey) [4].

Here we briefly present the main findings after 6 months of this enhanced surveillance.

## Description of cases

Seventy-five paediatric cases with severe conditions suspected to be potentially associated with EV infections were reported. Sixteen of them were excluded because of other aetiologies or lack of evidence of a possible EV infection and 59 were included in the analysis. Four cases in adults were also reported, however, here we only describe the 59 paediatric cases.

### Patient characteristics and symptoms

Median age at symptoms onset was 3 years, ranging from 1 month to 15 years, and the male to female sex ratio was 1.2 (32 male vs 27 female). Two thirds of the patients lived in the Paris area (Ile de France, n = 38) and 17% in Auvergne-Rhône-Alpes (n = 10), representing respectively 20% and 15% of the French metropolitan population, while further six of the 13 French metropolitan regions reported only 1 to 2 cases each.

The dates of symptoms onset ranged from 16 May to 30 October 2016 (week 20 to week 43), see Figure.

**Figure:**
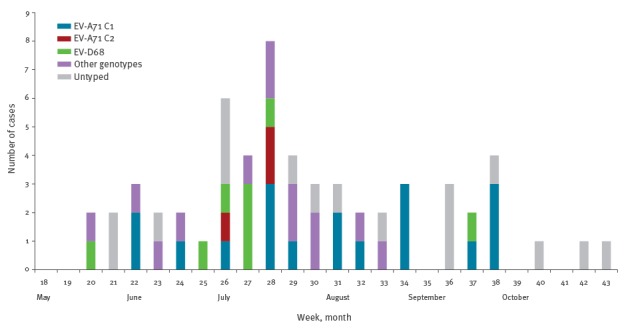
Distribution of severe paediatric cases linked with EV infections over time, by enterovirus genotype and week of symptoms onset, Metropolitan France, May–October 2016 (n=59)

EV: enterovirus.

Fifty-two of 59 children (88%) presented with severe acute neurological symptoms such as rhomboencephalitis (n=15), encephalitis (n=11), cerebellitis (n=5), myelitis (n=4), cranial nerve radiculitis (n=2); 13 other cases had combined neurological disorders and two children had both severe rhomboencephalitis and myocarditis; three had severe neonatal sepsis and four presented with isolated cardiac symptoms i.e. myocarditis, pericarditis, acute cardiac failure.

One patient died and 21 patients needed prolonged hospital stay because their condition was severe and/or they had persistent myocardial or neurological deficits, such as polio-like persistent peripheral neuropathy, at hospital discharge. At least four children needed prolonged ventilator support (through a tracheostomy) and/or feeding support after being admitted in a rehabilitation center, because of persistent brain stem dysfunction.

### Enterovirus detection and clinical picture associated with genotypes identified

In only eight of the 59 cases (13.5%), EV detection by real-time (RT)-PCR (5’ untranslated region) on CSF samples was positive, while EV was detected in most peripheral samples, e.g. in stools of 43 (73%) and/or nasopharyngeal aspirates of 37 (62.7%) cases. In 42 cases, the EV could be typed (71%) by sequencing of the viral protein (VP)1 or the VP4-VP2 coding gene, performed by the NRLs.

EV-A71 was identified in 21 of 42 cases (50%); 18 EV-A71 belonging to the subgenotype C1, one associated with EV-D68, and three EV-A71 C2 among which one associated with an echovirus (E)3. All cases presented with rhomboencephalitis, encephalitis or encephalomyelitis, except one fatal case of acute cardiac failure. For the latter a brain stem failure cannot be excluded as brain MRI could not be performed before death. Two cases had presented with a hand, foot and mouth disease before developing neurological symptoms.

Excluding the EV-D68 case which was associated with EV-A71 C1 described above, EV-D68 was identified in eight of 42 cases. Four of them presented with neurological disorders i.e. rhomboencephalitis or myelitis, three with cardiac symptoms i.e. myocarditis, pericarditis, acute cardiac failure, and one with neonatal sepsis.

In the remaining 13 cases, various EV were identified: coxsackie (CV) A6 (n = 2), CV-A16, CV-A10 and human rhinovirus (HRV) A56, CV-B3 (n = 2), CV-B2, CV-B5, E-25 (n = 2), E-13, E-6v, and E-30. All of the cases infected presented with neurological disorders.

## Discussion

In France, routine EV surveillance and molecular typing involve the RSE network and focus mainly on EV-associated neurological symptoms in hospitalised patients, as one of the main aims of this surveillance is to confirm the absence of circulation and to detect any possible importation of poliovirus, in a timely manner. Through this network, only a few sporadic cases of meningoencephalitis linked to EV-A71 [5-7] and one case of acute flaccid paralysis linked to EV-D68 [8] have been described in the country in the past 15 years. The impact of EV-A71 may have been previously underestimated because stool and respiratory specimens were not systematically collected from patients. Still, the enhanced surveillance set up in 2016 yielded an unusual number of reports of severe paediatric neurological cases associated with EV-A71. Moreover, whereas EV-A71 subgenogroup C2 viruses had been predominant in France since 2006 [5,9], in 2016, the EV-A71 subgenogroup C1 viruses were predominant. These EV-A71 C1 viruses had not previously been detected in France (data not shown) and were closely related to a new cluster of EV-A71 C1 viruses detected in 2015 in Germany [10,11]. Most of the cases were diagnosed in late July, concurrently with the usual peak of EV circulation in the country, but cases were still identified in September and October. Other EVs circulated scattered over the 6-month period of study, especially EV-D68, that circulated mainly in July, but one case was still detected in September.

Taking into account the severity of the initial and persisting symptoms and the fact that EV-A71 is the most neuropathogenic non-polio enterovirus in humans [5], it has been decided to prolong the enhanced surveillance at least until the end of 2016, as another peak in EV circulation may be observed during this autumn.

Ascertaining the diagnosis of EV infection was difficult during this outbreak, especially when investigating cases retrospectively. While EV detection in CSF samples was mostly negative, clinicians had to change their practise and, following the NRLs’ recommendations, ask for EV detection in respiratory samples and rectal swabs or stool specimens. Such specimens, however, were not systematically available from severe neurological cases before surveillance was reinforced. Therefore, virological information was better for prospectively reported cases. The input of the RSE was an important complementary source of information, allowing rapid reporting of several cases that would have been missed otherwise. Nevertheless, case reporting was probably not exhaustive. Another remaining challenging question is the pathophysiology of such severe conditions in this paediatric population, in which asymptomatic or mild EV infections are very common, raising hypotheses concerning special strain virulence or peri-infectious inadequate immune response, as is often the case in paediatric non necrotising encephalitis.

## Conclusion

Without control measures other than strengthening of personal hygiene for close contacts, and because of disease severity, accurate diagnosis of EV-associated severe conditions is a key issue. Clinicians should be encouraged to obtain stool and respiratory specimens from all patients presenting with symptoms suggestive of severe neurological disorders such as encephalitis, rhombencephalitis, cerebellitis, acute flaccid myelitis or acute flaccid paralysis, for EV detection and characterisation. Furthermore, the known epidemiological pattern and clinical picture of both EV-A71 and EV-D68 may be changing in Europe, as shown by the recent outbreak of EV-A71 in Spain, or the clusters of EV-D68 infections recently reported by Scotland and Sweden, making it necessary to reinforce the vigilance towards those infections.
